# Genetically-directed Sparse Neuronal Labeling in BAC Transgenic Mice through Mononucleotide Repeat Frameshift

**DOI:** 10.1038/srep43915

**Published:** 2017-03-08

**Authors:** Xiao-Hong Lu, X. William Yang

**Affiliations:** 1 Center for Neurobehavioral Genetics, Semel Institute for Neuroscience & Human Behavior, Los Angeles, CA 90095, USA; 2 Department of Psychiatry & Biobehavioral Sciences, David Geffen School of Medicine, University of California at Los Angeles, Los Angeles, CA 90095, USA

## Abstract

Mosaicism with Repeat Frameshift (MORF) allows a single Bacterial Artificial Chromosome (BAC) transgene to direct sparse labeling of genetically-defined neuronal populations in mice. The BAC transgene drives cell-type-specific transcription of an out-of-frame mononucleotide repeat that is placed between a translational start codon and a membrane-bound fluorescent protein lacking its start codon. The stochastic frameshift of the unstable repeat DNA in a subset of BAC-expressing neurons results in the in-frame translation of the reporter protein hence the sparse neuronal labeling. As a proof-of-concept, we generated D1-dopamine receptor (D1) BAC MORF mice that label about 1% striatal D1-expressing medium spiny neurons and allow visualization of their dendrites. These mice enable the study of D1-MSN dendrite development in wildtype mice, and its degeneration in a mouse model of Huntington’s disease.

One major challenge to study the mammalian brain is to visualize and perturb genetically-defined neurons at single-cell resolution. Several genetic methods have been developed to achieve sparse labeling of single neurons, enabling the study of the relationship between neuronal morphology and neuronal function or dysfunction^1^,^2^. These current methods, however, have certain limitations^2^. For example, transgene promoter silencing due to random chromosomal insertions (e.g. Thy1 promoter transgene) is not readily applicable to other promoters to label diverse cell types. Also, low-efficiency and inducible site-specific recombination based methods may require the generation and crossing of multiple transgenic mouse lines with labeling frequency that may be variable and difficult to control.

Recent advances in transgenic or genome targeting technologies provide reliable genetic methods to express reporter genes (e.g. fluorescent proteins) in endogenous-like patterns in specific neuronal cell types in mice^1^,^3^. However, such genetic labeling in a densely packed neuronal cell population often precludes the visualization of their detailed morphology (i.e. dendrites and axons). Thus, a simple method is still lacking to readily generate transgenic mice, based on genetic constructs with proven neuronal cell-type specificity, but substantially reduced labeling frequency to confer sparse and stochastic (random) labeling of defined neurons for morphological analyses.

Microsatellite repeats (e.g. mono-, di- or tri-nucleotides) are ubiquitous but non-randomly distributed across all genomes and are prone to frameshift mutations^4^,^5^. The frequency of such frameshifts may be dependent on repeat type, length, and other cell-type-specific factors (e.g. mismatch repair efficacy)^6^. In rare cases, microsatellite repeats exist in the coding regions of genes and may cause neurological disorders and cancer when frameshift occurs^5^,^7^. To date, there are a few mouse genetic studies that take advantage of mononucleotide repeat frameshift to study frameshift mutation rates *in vivo*^8^,^9^,^10^, or inducing mosaic Cre expression in intestinal cells^11^,^12^. These studies did not reveal the potential of this approach for genetically directed sparse neuronal labeling, because the frameshift rate for G_11_ repeat in the brain is about 10^−4^–10^−5^ (Refs 8, 9, 10), which is much lower than the desirable sparse labeling frequency of about 1% neurons as in the classical Golgi method^2^.

## Results

In this study, we reasoned that a long mononucleotide repeat (i.e. G_22_) might be combined with a proven neuronal cell-type-specific genetic construct such as a BAC transgene to achieve the sparse neuronal labeling at a desirable frequency to allow single neuron morphological studies. As a proof-of-concept for the MORF strategy, we used the murine dopamine receptor D1 (Drd1a) BAC that drives transgene expression in the direct-pathway medium spiny neurons (i.e. D1-MSNs)^3^. This is one of two major striatal projection neuron cell types that mediate basal ganglia function in motor control and learning^13^. Dysfunction or degeneration of such neuronal pathways have been implicated in major neurological and psychiatric disorders including Huntington’s disease (HD)^13^.

**Figure 1:**
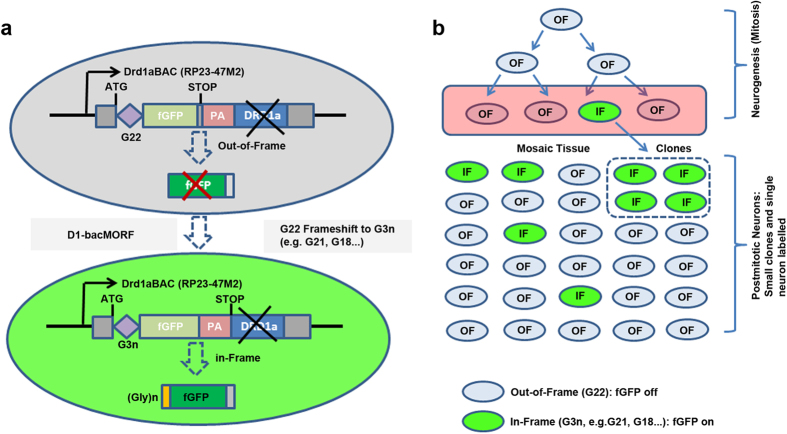
Illustration of the Mosaicism with Repeat Frameshift (MORF) Strategy for Genetically Directed Sparse and Stochastic Labeling of Single Neurons in the Mouse Brain. (**a**) A mononucleotide G_22_ repeat was inserted between translational initiation codon and farnesylated GFP (fGFP), with this cassette inserted into murine *Drd1a* BAC (*Drd1a*-bacMORF-fGFP). In D1-MSNs without G_22_ frameshift to G3n frame, fGFP is not expressed. Only neurons with frameshift of G_22_ to G_3n_ (e.g. 21, 18) will express fGFP with a small N-terminal polyglycine tag (translated from G3n). PA: Polyadenylation signal. (**b**) Schematics to illustrate that mitotic instability of the mononucleotide repeat during neurogenesis (mitosis) could render the out-of-frame (OF) G_22_ to undergo a frameshift mutation to allow the fluorescent marker protein to be reverted into in-frame (IF), and therefore the expression of the marker to label a small clonal lineage of cells. In some circumstances (e.g. damaged DNA undergoing mismatch repair), such IF reversion could happen in post-mitotic single neurons.

The original Drd1a-BAC-EGFP mice label the entire D1-MSN population precluding visualization of individual neuronal morphology^3^. To test the MORF strategy, we engineered the *Drd1a* BAC to insert a repeat of 22 deoxyguanines (G_22_) between the ATG initiation codon and a membrane-tethered farnesylated GFP (fGFP) lacking its own translation initiation codon (Fig. 1a). Due to the instability of the repeat sequence during neurogenesis or in postmitotic neurons (e.g. DNA repair, Fig. 1b), we expect that in a subset of BAC expressing neurons the repeat sequence will undergo a frameshift mutation in the G_22_ repeat from out-of-frame to in-frame, hence the cargo protein (fGFP) will be translated with a small N-terminal polyglycine tag resulting in genetic labeling of only a subset of the D1-MSNs.

**Figure 2:**
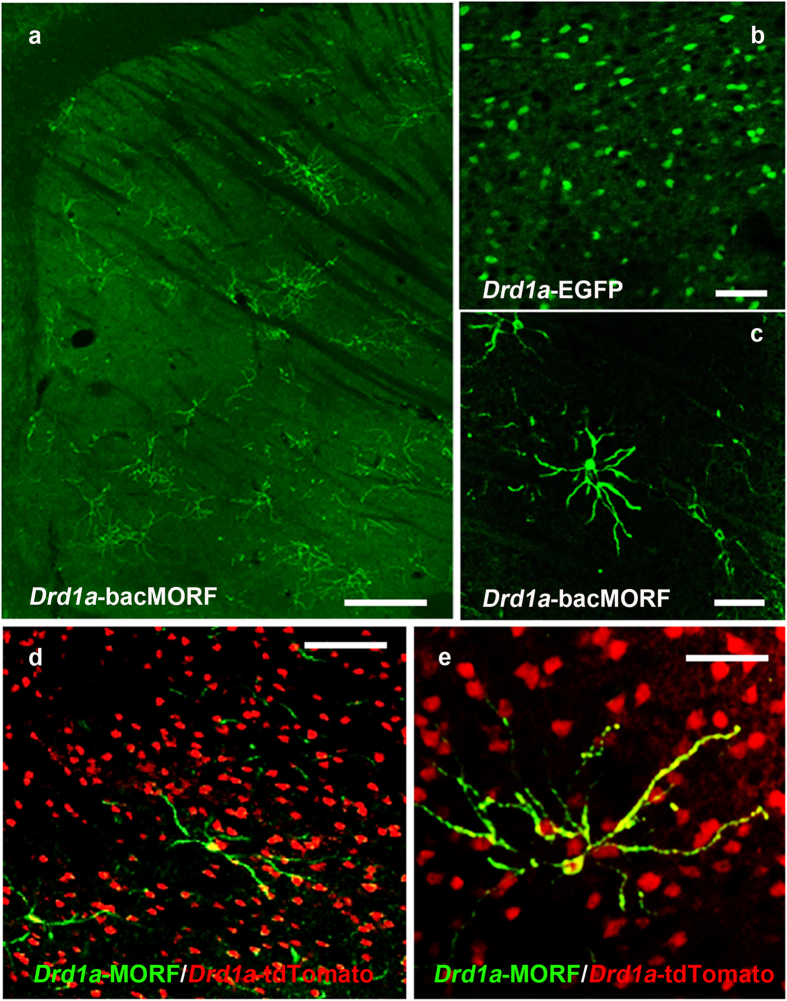
Sparse and Stochastic Labeling of Striatal Direct-pathway Medium Spiny Neurons Expressing Dopamine Receptor D1 (D1-MSNs). (**a** and **c**) Representative images of different magnifications to illustrate sparse and stochastic labeling of D1-MSNs in the striatum of *Drd1a*-bacMORF-fGFP mice (scale bar = 40 μm). (**b**)The expression pattern is distinct from that of GENSAT *Drd1a*-BAC-GFP mice that label all D1-MSNs but cannot reveal their dendritic morphological details (scale bar = 10 μm). In the *Drd1a*-bacMORF-fGFP and *Drd1a*-BAC-tdTomato double transgenic mice, *Drd1a*-bacMORF selectively and sparsely labels only D1-MSNs as indicated by the co-localization of fGFP (green) with tdTomato (red) labeling (scale bar in **d** = 20 μm; scale bar in **e** = 10 μm).

We performed pronuclear injections to create two *Drd1a*-bacMORF-fGFP (or *Drd1a*-bacMORF) founder mice, which we subsequently backcrossed and maintained as two lines (A and B line) on a C57BL/6J background. The transgenic mice were born at a Mendelian ratio and lack apparent behavioral phenotypes or pathology (data not shown). We quantified the transgene copy number and found A line has 3 copies and B line has one copy of the BAC transgene (Supplementary Fig. S1). We next performed GFP immunofluorescent staining of adult *Drd1a*-bacMORF striatum and found that sparse labeling of a subset of striatal neurons with characteristic MSN morphology in both lines (Fig. 2a and c, Supplementary Fig. S2), a pattern that is distinct from the GENSAT *Drd1a*-EGFP mice (Fig. 2b). The sparsely labeled neurons, which consisted of single neurons or small cluster of neurons (likely clones), are located in both dorsal and ventral striatum, and their dendritic structures can be clearly visualized (Fig. 2). The distribution of the labeled single or small cluster of neurons appears random throughout the striatum, consistent with stochastic labeling of the MORF method. Since the fluorescent signal for the labeled neurons is not bright enough for direct imaging, immunostaining was necessary for optimal visualization (see Methods). To confirm that the labeled MSNs are indeed D1-MSNs, we crossed *Drd1a*-bacMORF mice with *Drd1a*-BAC-tdTomato mice^14^, which selectively express tdTomato in D1-MSNs. The double transgenic mice showed that all of the sparsely labeled neurons in *Drd1a*-bacMORF mice are also positive for tdTomato (Fig. 2d,e), demonstrating the sparse labeling of D1-MSNs in the *Drd1a*-bacMORF mice. Importantly, quantification of the double transgenic mice showed the D1-MSN-specific labeling efficiency of about 0.978 +/− 0.586% in these mice.

**Figure 3:**
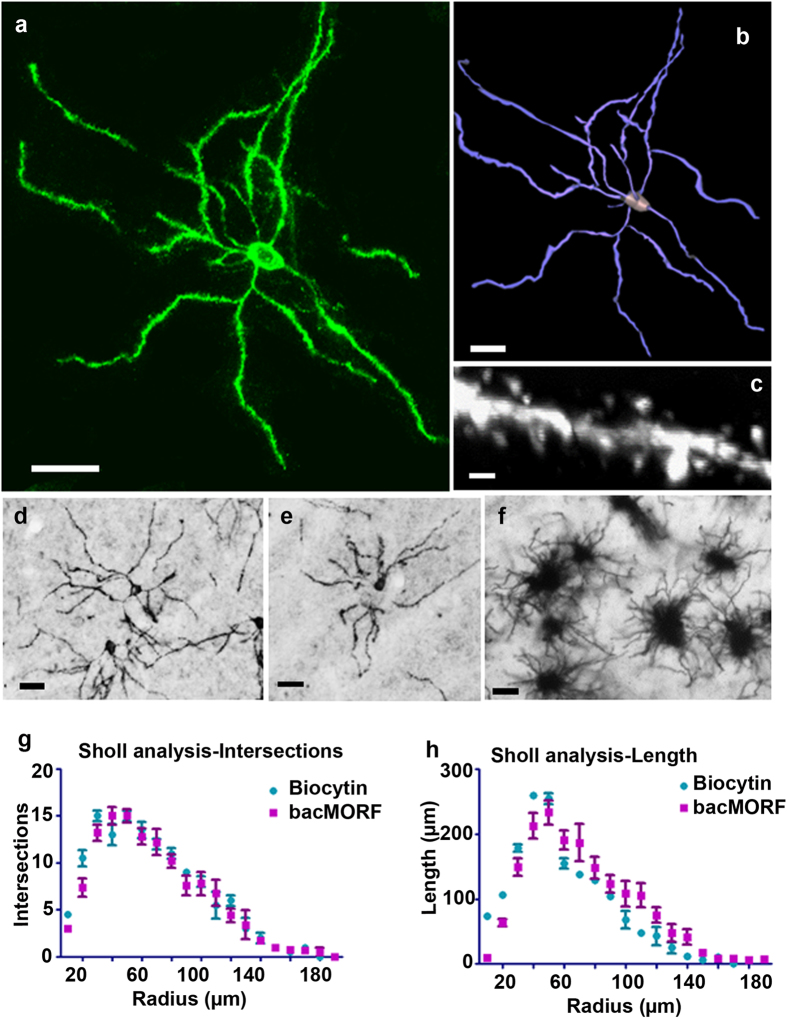
Imaging and 3D Reconstruction of Detailed Dendritic and Spine Structures in Single Labeled D1-MSNs in *Drd1a*-bacMORF-fGFP Mice. The MORF method can be used to visualize the dendritic structures of D1-MSNs. (**a**) A projection of stacked confocal images of a single striatal D1-MSN labeled by the MORF method (scale bar = 10 μm). (**b**) A 3D reconstruction of the dendrite and spine structures using Neurolucida. (**c**) A projection of stacked confocal images of the dendritic spines from the D1-MSN depicted in 3A. Structures of spines can be visualized from the images (scale bar = 1 μm). (**d**–**f**) A comparison of the immunohistochemical staining of striatal D1-MSNs labeled by MORF ((**d**,**e**) scale bar = 10 μm) and MSNs labeled by the Golgi method ((**f**) scale bar = 10 μm). Striatal MSNs stained by the Golgi method (**f**) often are clustered with overlapping dendritic structures from multiple MSNs. Immunostaining for GFP in *Drd1a*-bacMORF-fGFP mice (**d** and **e**) show the labeling of small clusters (**d**) or single (**e**) D1-MSNs with a detailed dendritic structure for individual labeled neurons. (**g**,**h**) Quantitative comparison of the immunohistochemical staining of striatal D1-MSNs labeled by MORF and by biocytin microinjection into D1-MSNs in GENSAT *Drd1a*-BAC-GFP mice. Neurolucida and NeuroExplorer Suites (MicroBrightField) were used for 3D reconstruction of the dendritic structures of D1-MSNs labeled by the two methods, and for quantitation using Sholl analysis for intersections (branching) and dendritic length. Two-way ANOVA factored with labeling methods and distance to cell body could not detect significant difference between two methods (n = 5 for each genotype; Length: F = 0.992, P = 0.352; Length: Intersection: F = 2.047, P = 0.196).

The incorporation of the membrane-tethered fGFP in the MORF method should facilitate the visualization and reconstruction of fine anatomical structures such as the dendrites for 3-dimensional (3D) reconstruction. Indeed, immunofluorescent staining in *Drd1a*-bacMORF mice revealed the details of dendritic architecture that can be readily reconstructed in 3D using Neurolucida (MicroBrightField; Fig. 3a,b), which in turn allows quantitative morphological analyses. Moreover, the MORF method also allows visualization of at least a subset of dendritic spines on the labeled D1-MSNs (Fig. 3a,c). Importantly, immunohistochemical detection of MORF-labeled neurons (Fig. 3d,e) appears to show better resolution of single labeled MSNs compared to the traditional Golgi staining, which shows more clustered MSNs in the striatum (Fig. 3f). We next examined axonal labeling in *Drd1a*-bacMORF striatum, and found only the proximal axonal segments of D1-MSNs can be visualized upon immunofluorescent staining (Supplementary Fig. S3).

Finally, to quantitatively assess whether MORF-labeled D1-MSNs reveal comparable dendritic architecture compared to those labeled using a traditional method, we performed 3D reconstruction and Sholl analyses of the dendritic trees of D1-MSNs labeled by *Drd1a*-bacMORF mice and those with biocytin microinjection in *Drd1a*-BAC-GFP mice (Fig. 3g,h). The comparison did not reveal any significant differences in dendritic branching patterns and length between D1-MSNs labeled by the two methods. This result showed not only that the MORF method can reveal detailed dendritic structures of D1-MSNs, but also that membrane-tethered GFP with its N-terminal polyglycine tag (encoded by G_3n_ repeat) does not appear to be toxic to or alter the dendritic morphology of labeled neurons.

The endogenous Drd1a receptor is expressed in other neuronal populations, such as hippocampal pyramidal neurons and dentate gyrus granule cells, therefore we examined these cells. Indeed, we found individually labeled hippocampal pyramidal neurons and dentate gyrus granule neurons (Supplementary Fig. S4) in *Drd1a*-bacMORF mice. Thus, our study demonstrates that the MORF method can confer genetically directed sparse neuron labeling in multiple neuronal cell types in the brain.

**Figure 4:**
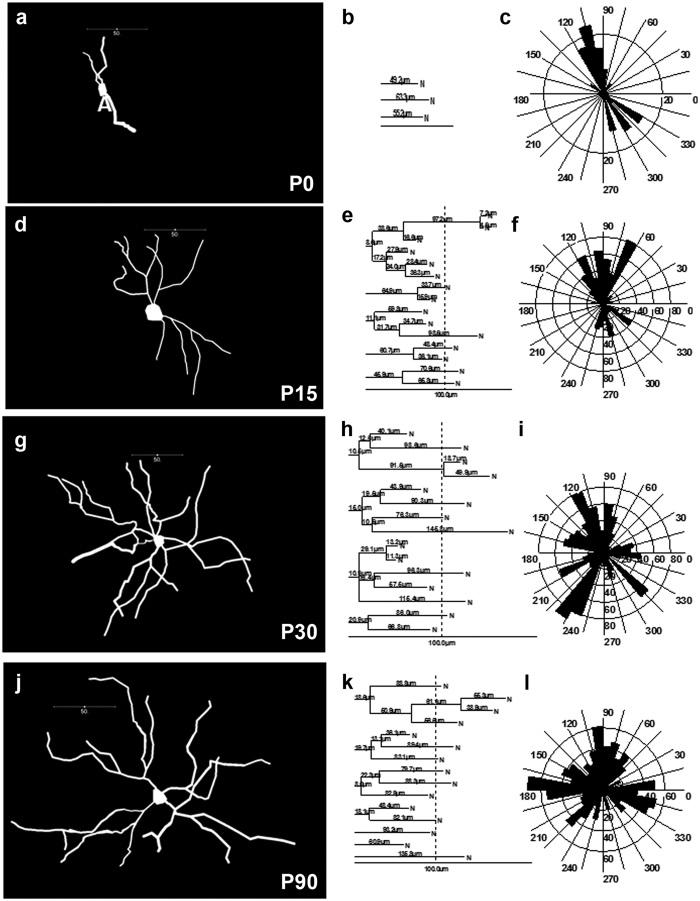
Using *Drd1a*-*bac*MORF Mice to Image Postnatal Dendritic Development in Striatal D1-MSNs. The MORF method reveals the postnatal growth of dendritic arbors in single D1-MSNs at postnatal day 0 (**a–c**), 15 (**d–f**), 30 (**g–i**) and 90 (**j–l**). Individual labeled neurons were 3D reconstructed using Neurolucida (**a**,**b**,**c**,**d**), which were then used for Dendrogram (**e**,**f**,**g**,**h**) and Sholl Analyses (**i**,**j**,**k**,**l**) to obtain quantitative morphological data for the D1-MSNs at these different ages.

To demonstrate the utility of the MORF method to study neurodevelopment, we next examined postnatal changes in dendritic structures of the sparsely labeled D1-MSNs. Although prior studies using Golgi or dye injection methods have shown that striatal MSNs undergo extensive postnatal dendritic growth^15^,^16^,^17^, no prior study has examined postnatal dendritic development of identified D1-MSNs. We imaged and reconstructed detailed dendritic structures of labeled D1-MSNs in the MORF mice at postnatal days 0, 14, 30 and 90 (Fig. 4). We performed dendrogram (Fig. 4b,e,h,k) and Sholl analyses (Fig. 4c,f,i,l) of the dendritic branching patterns of multiple reconstructed D1-MSNs at each age (N > 5 per age). Our study reveals that the dendritic arbors of D1-MSNs undergo robust growth in terms of branching points, length, and diameter from birth to P30, with relatively modest dendritic growth being seen between P30 and P90. Our study demonstrates the utility of the MORF method to study dendritic development in a genetically-defined neuronal cell type *in vivo*.

A potentially powerful application for genetically directed sparse neuronal labeling is to study neuropathology, such as neurodegeneration, at single cell resolution. Mouse models of neurodegenerative disorders often exhibit partial disease phenotypes (e.g. neuronal atrophy) but often lack frank neuronal loss^18^,^19^,^20^. In Huntington’s disease (HD), the striatal MSNs in both the direct and indirect pathways undergo massive cell loss^21^. In mouse models of HD, such as the heterozygous zQ175 knockin mice expressing full-length endogenous murine mutant Huntingtin (mHtt), recapitulate the progressive striatal MSN dysfunction measured by electrophysiology^22^,^23^ and transcriptome profiling^24^. However, the zQ175 mice do not show significant striatal MSN loss by 12 months of age^24^,^25^. Although neurodegeneration in such models can manifest partially as degeneration of neuronal processes, the use of traditional methods, such as Golgi staining or biocytin microinjection into MSNs, did not reveal any significant MSN dendritic pathology at 7 and 12 months of age in zQ175 mice^23^,^26^. However these methods could not distinguish MSN subtypes (D1- vs D2-MSNs), hence it is unclear if examining MSN subtypes could reveal evidence of dendritic pathology in this HD mouse model.

**Figure 5:**
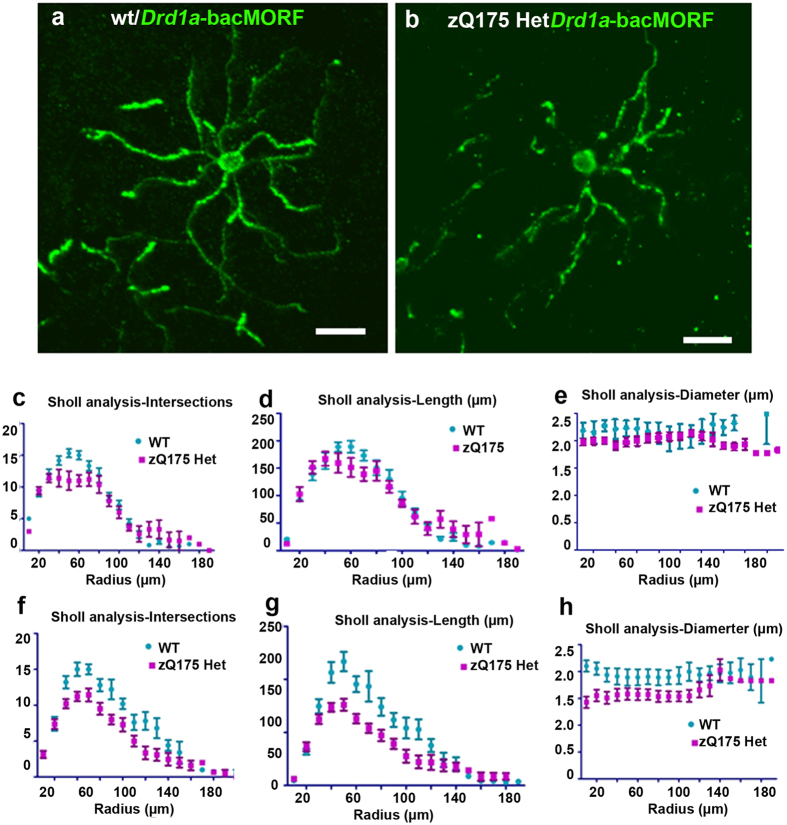
Using *Drd1a*-*bac*MORF Mice to Visualize Dendritic Pathology in an Huntington’s Disease Knock-in Mouse Model. *Drd1a*-bacMORF mice were crossed with an HD knock-in mouse model expressing full-length mHtt with 175Q from its endogenous locus. Single labeled D1-MSNs in both wildtype and zQ175 heterozygous mice were examined at 1–2 months and 6–7 months of age, reconstructed using Neurolucida, and subsequently quantified using Sholl analysis. (**a**) D1-MSNsin wildtype mice at 6–7 m of age. (**b**) An example of MORF-labeled D1-MSN with aberrant dendritic pathology in zQ175 mouse striatum at 6–7 m of age. (**c–e**) Scholl analyses of reconstructed D1-MSNs at 1–2 m of age do not show significant difference in dendritic branching pattern (i.e. intersection), length or diameter between wildtype and zQ175 heterozygous mice (two-way ANOVA, p > 0.05, (**c**,**d**,**e**)). (**f–g**) There is a statistically significant reduction of dendritic branching, length and dendrite diameter in MORF-labeled D1-MSNs in zQ175 heterozygous mice at 6–7 months of age compared to wildtype mice at the same age (two-way ANOVA, p < 0.05).

We reason that the use of *Drd1a*-bacMORF mice to selectively visualize D1-MSN dendrites may provide a simple and potentially sensitive method to detect early, MSN-subtype specific dendritic pathology in an HD mouse model. We crossed *Drd1a*-bacMORF mice to zQ175 heterozygous mice (in C57BL/6 background), and then analyzed double transgenic mice carrying *Drd1a*-bacMORF and zQ175 heterozygous alleles as well as *Drd1a*-bacMORF controls at presymptomatic age (1–2 m) or a symptomatic age (6–7 m) with motor deficits but no prior known neuropathology^22^,^25^. We imaged and reconstructed MORF-labeled D1-MSNs in the striata of these mice and quantified the dendritic parameters using Sholl analyses (Fig. 5). At the pre-symptomatic age, we found that dendritic branches, length and diameters of the labeled D1-MSNs were comparable between zQ175 and control mice (Fig. 5c–e, N = 12 per genotype from three littermates at the same age, for genotype difference, intersections: F = 0.288, P = 0.597; Length: F = 0.288, P = 0.597; Dendritic diameter: F = 0.002, P = 0.967). Interestingly, at an symptomatic age of 6-7 months, we found a statistically significant reduction of dendritic branching, length and diameter in the MORF labeled D1-MSNs in zQ175 striata compared to those from wildtype controls (Fig. 5f–h, N = 15 per genotype from three littermates at the same age, for genotype difference, intersections: F = 20.549, P < 0.05; Length: F = 26.828, P < 0.01; Dendritic diameter: F = 21.247, P < 0.05). To our knowledge, this is the first report of MSN subtype-specific dendritic pathology at such an early age in a full-length mHtt expressing mouse model. Moreover, unlike some traditional single-cell labeling methods that are incompatible with double immunofluorescent staining (e.g. Golgi), we showed MORF method can be readily combined with a second antibody to detect additional HD-like pathology such as mHtt aggregation (Supplementary Fig. S5). Future studies would be interesting to examine the relationship of such molecular pathology to dendritic phenotypes in D1-MSNs of HD mice.

## Discussion

In summary, we describe here a conceptually novel method (MORF), based on the mononucleotide repeat frameshift mechanism, to enable the use of a single BAC transgene, with proven neuronal cell-type promoter activity *in vivo*, to sparsely and stochastically label genetically-defined neurons for detailed morphological studies. As a proof-of-concept study, we showed the MORF method allows a Drd1 BAC transgene to express a membrane-bound fluorescent maker protein in about 1% of D1-MSNs, which allows visualization of their dendritic architecture, development, degenerative pathology in an HD mouse model. Importantly, the latter study, to our knowledge, is the first report of MSN subtype-specific dendritic pathology in a full-length mHtt expressing HD mouse model. At least in this case, MORF is proven to be more sensitive to detect such pathology than the traditional Golgi staining or biocytin microinjection approaches in the same HD mouse model^23^,^26^.

In principle, the MORF reporter can be used in conjunction with other defined genetic promoters (e.g. BAC transgenes or knock-in to endogenous loci) to achieve sparse labeling of genetically-defined cell types, but future studies are needed to thoroughly evaluate the broad utility of such an approach. Since the frequency of mononucleotide repeat frameshift depend on repeat types (e.g. G_n_ and C_n_ repeats have higher frequencies than A_n_ or T_n_ repeats) and length^4^,^6^,^27^, the test of additional repeat types and lengths may also expand the applications of MORF technology to achieve different labeling efficiencies for optimal studies of different neuronal populations. Finally, strategies to enhance the MORF reporter signals, such as the use of stronger promoters, transcriptional amplification mechanisms^28^, and reporters with enhanced detection signals^29^, may improve the use of MORF to visualize more complete morphology of labeled single neurons. Ideally, it would include synaptic structures, dendritic trees, and brain-wide axonal projections. In conclusion, our study provides important proof-of-concept for a novel genetic approach to label and study the morphological details of genetically-defined single neurons, which should facilitate the study of how neuronal morphology may be causally linked to normal function, or dysfunction and degeneration in the mammalian brain.

## Methods

### Generation of *Drd1a*-bacMORF-fGFP BAC Transgenic Mice

A 200 kb murine Dopamine Receptor 1 (Drd1a) BAC (RP23-47M2) used in GENSAT^3^ was obtained from the BACPAC resource center (Oakland Children’s Hospital, Oakland). The transgene that contains the mononucleotide tract (G_22_), followed by a farnesylated-GFP coding and polyA region was inserted into exon 1 of Drd1a preceding the endogenous translation initiation codon (Fig. 1a). Two oligonucleotides were synthesized and annealed to generate G_22_ mononucleotide tract (Oligo1: 5′-CTCGAGGCCACCATGGGGGGGGGG GGGGGGGGGGGGGAAGCTT-3′; Oligo 2: 5′: AAGCTTCCCCCCCCCCCCCCCCCCCCCCCATGGTGGCCTCGAG -3′) flanked by two restriction enzyme (XhoI and HindIII) sites to create a frame shift mutation for farnesylated GFP. Maxiprep DNA was prepared from the modified Drd1a BAC and purified through cesium prep. The integrity of the BAC DNA was examined on a pulsed-field gel according to an established protocol^30^. The intact BAC DNA (1 ng/ul) was microinjected into fertilized F1 mouse (C57BL6/CBA) zygotes to generate BAC transgenic mice. Two of the D1-MORF BAC transgenic lines, designated lines A and B, were subsequently germline transmitted and expanded by crossing with wildtype C57BL/6 mice. The heterozygous transgenic mice and their wildtype littermates were used for the subsequent studies. Mouse care in the current study was in accordance with the United States Public Health Service Guide for the care and Use of Laboratory Animals. The procedures were approved by Chancellor’s Animal Research Committee (ARC) at UCLA. Veterinarian care was provided by the UCLA Division of Laboratory Animal Medicine. Animals were housed in a specific-pathogen-free barrier facility at UCLA, with up to four mice per cage with food and water available *ad libitum*. They were housed in a temperature-controlled environment with 12 hour light/dark cycle.

### Transgene copy number determination by quantitative PCR

Tail genomic DNA was extracted using phenol-chloroform (Sigma-Aldrich) with ethanol precipitation. Quantitative PCR was performed using the KAPA SYBR Fast qPCR mix with primer sets targeting the 3′UTR in Exon of Drd1a (For: AAA GTT CCT TTA AGA TGT CCT; Rev: TGG TGG CTG GAA AAC ATC AGA) and the housekeeping gene HPRT (Forward: GCT CGA GAT GTC ATG AAG GAG A; Reverse: TCA GTG CTT TAA TGT AAT CCA GC) as reference for normalization. PCR reactions were carried out on a Roche LightCycler 480 with the following cycling conditions for all primer sets: 95 °C for 3 min and then 40 cycles of 95 °C for 15 sec, 56 °C for 25 sec, and 72 °C for 1 sec, followed by a melting curve analysis and 40 °C for 10 sec. All samples were run in three replicates for each primer set. Using the 2^−ΔΔCt^ relative expression method^31^, the average CT values for the three replicates of each gene was normalized to the average CT values for the housekeeping gene (HPRT) to calculate the expression level of Drd1 in the transgenic mice relative to WT mice.

### Immunofluorescence and Imaging

*Drd1a*-bacMORF transgenic mice were perfused transcardially with 4% paraformaldehyde in 0.1 M phosphate buffered saline (PBS). Tissues were isolated and fixed in 4% PFA in 0.1 M PBS at 4 °C overnight and cryoprotected for >24 hours in 30% sucrose in PBS, then embedded in Tissue-Tek OCT (VWR, Cat. No 25608-930) prior to cryostat sectioning. Tissues were sectioned at 20 μm on a Leica 1800 Cryostat (Deerfield, IL) and cryoprotected for further usage. Floating sections were immunostained in 24-well plates using the procedures below and subsequently mounted on slides. For immunofluorescence, cryosections were washed three times for 10 min in PBS, blocked with 10% normal goat serum (GS) in PBS + 0.3% Triton X-100 (PBT) for 1 hour at room temperature, and stained at 4 °C overnight with primary GFP antibodies in 5% GS in PBT (1:500 dilution). Following four washes for 10 min in PBT, sections were stained for 1 hour with secondary antibodies. The sections were washed four times for 10 minutes with PBT, treated with DAPI (Sigma, Cat. No. D8417), rinsed for 10 minutes in PBS, and mounted in Fluoro-Gold mounting medium. Some synapse images were taken with immunofluorescence staining with the standard Tyramide Signal Amplification (TSA, Perkin Elmer) protocol.

For imaging D1-MORF labeled axons, 60 μm thick sagittal brain sections were cut and washed in 0.01 M PBS three times, each for 10 min. The sections were blocked with 10% GS in PBS with 0.3% Triton X-100 (PBT) for 1 hour, then incubated in PBT with rabbit anti GFP antibody (1:5000, Life Tech) for 48 hours at 4 °C. Following four washes for 10 min each in PBT, sections were stained for 2 hours with goat anti-rabbit antibody (1:1000, Vector Laboratories) at room temperature or overnight at 4 °C with shake. The sections were then washed four times with PBT for 10 minutes each, processed with TSA amplification, and subsequently incubated in Alexa Fluor^®^ 594-streptavidin (1:500, Vector Laboratories). Images were taken using an LSM510 confocal system (Carl Zeiss Inc., Oberkochen, Germany) or a Zeiss fluorescence microscope with a CCD camera and processed using LSM software (Zeiss) and ImageJ.

### 3D Neuronal Reconstruction and Quantification of Dendritic Morphology

To visualize the refined structures of D1-MSNs, Neurolucida (MicroBrightField, Williston, Vermont) was used to trace dendritic arbors of MSNs three-dimensionally and to mark spines. A cell was rejected if the soma was not intact or if it displayed discontinuity of processes, suggesting compromised integrity. Serial optical sections (Z-stacks) were acquired on a laser-scanning confocal microscope (LSM 510; Zeiss) with a 40×/1 NA objective (Zeiss). Images were stored at 8-bit image depth at a resolution of 512 × 512 pixels (0.22 × 0.22 × 1 μm). Z-series of the same cell were stitched together and subsequently reconstructed and analyzed using the MicroBrightField Neurolucida/Neuroexplorer suite.

### Statistical analysis

All statistical analyses were performed using the SPSS statistics (Version 22.0, IBM). For each of the basic morphometric parameters (intersections, length, and dendritic diameter), we tested for significant differences between genotype (H0: a given parameter does not significantly differ across distance from the soma). Each measurement was first tested for goodness of fit to a normal distribution within each age group using the Lilliefors test (α = 0.05). Where normality could be assumed, a student’s t-test or one-way analysis of variance (ANOVA) was used to test for differences across different groups, followed by a Least Significant Difference (LSD) post-hoc multiple-comparison test to test for specific differences between groups (α = 0.05). Where normality could not be assumed, a Mann-Whitney U test or Kruskal–Wallis non-parametric ANOVA was used to test for differences across groups followed by post-hoc multiple comparisons t-tests (LSD-adjusted, α = 0.05). Outliers were detected using the Explore function in SPSS and excluded form analysis. For Sholl analyses in zQ175 mice, either total dendrite length or total number of intersections within each shell was compared using a two-way ANOVA, with genotype and distance from the soma as factors (H0: a given parameter does not significantly differ across distance from the soma).

## Additional Information

**How to cite this article:** Lu, X.-H. and Yang, X. W. Genetically-directed Sparse Neuronal Labeling in BAC Transgenic Mice through Mononucleotide Repeat Frameshift. *Sci. Rep.* **7**, 43915; doi: 10.1038/srep43915 (2017).

**Publisher's note:** Springer Nature remains neutral with regard to jurisdictional claims in published maps and institutional affiliations.

## Supplementary Material

Supplemental Information
