# Supplementary material for: Genetically-directed Sparse Neuronal Labeling in BAC Transgenic Mice through Mononucleotide Repeat Frameshift
Source: Sci Rep. 2017 Mar 8;7:43915. doi: 10.1038/srep43915 (PMC5341054; doi:10.1038/srep43915)
Supplement: Supplemental Information [file srep43915-s1.docx]

**Supplementary Figures**

**Genetically-directed Sparse Neuronal Labeling in BAC Transgenic Mice through Mononucleotide Repeat Frameshift**

Xiao-Hong Lu^1,2, #^ and X. William Yang^1,2 *^

^1^Center for Neurobehavioral Genetics, Semel Institute for Neuroscience & Human Behavior, Los Angeles, CA 90095; ^2^Department of Psychiatry & Biobehavioral Sciences, David Geffen School of Medicine, University of California at Los Angeles, Los Angeles, CA 90095

* Correspondence: [xwyang@mednet.ucla.edu](mailto:xwyang@mednet.ucla.edu)

^#^ Present address: Department of Pharmacology, Toxicology and Neuroscience, Louisiana State University Health Sciences Center, Shreveport, LA, 71130

**
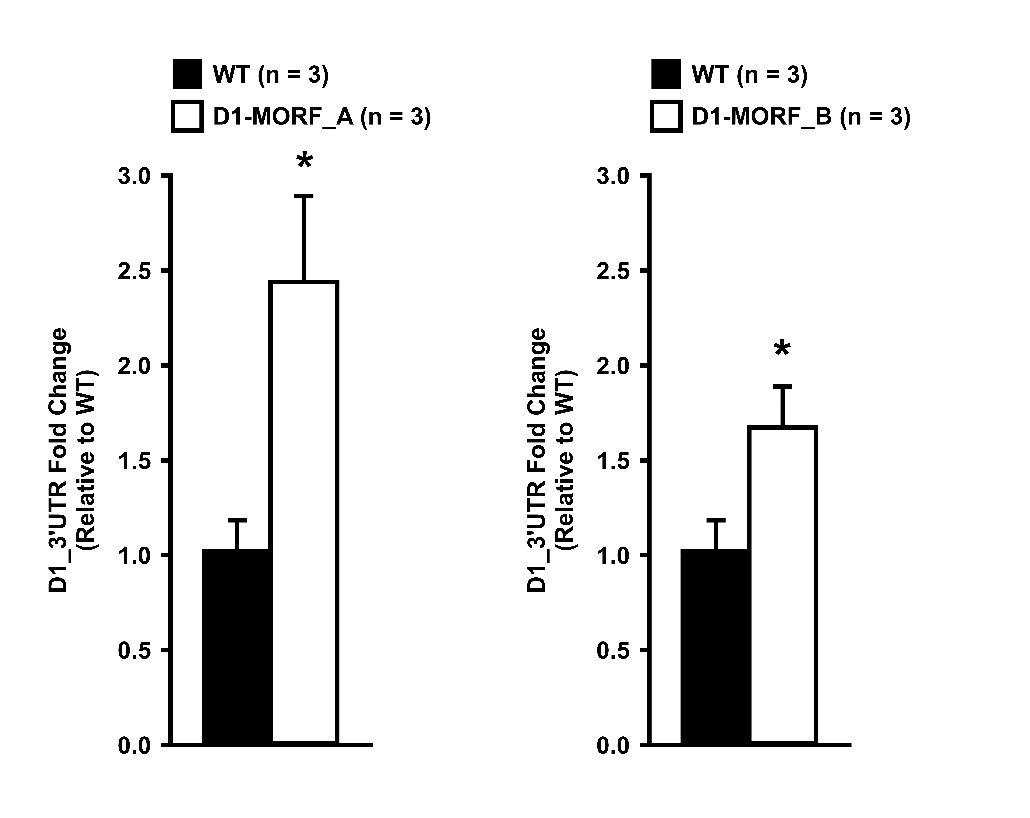
**

**Supplementary Figure S1. Quantitative PCR analyses of the genomic DNA to determine transgene copy numbers in two lines of D1-bacMORF-fGFP mice.** Primers specific for the 3’UTR in Exon 2 of the endogenous murine *Drd1a* gene were used to quantify transgene copy numbers in D1-bacMORF-fGFP mice. Mouse wildtype genomic DNA was used as a two copy per diploid genome control. Quantitative PCR (qPCR) of purified tail genomic DNA (gDNA) from D1-bacMORF-fGFP lines A and B and WT controls (n=3 per genotype) was performed with these Drd1a-specific primers. Relative quantification using the housekeeping gene, Hprt, as the reference gene for normalization showed that line A contains 3 extra copies of the Drd1a genomic locus sequences (i.e. BAC transgene copies) while line B has one extra copy compared to that of WT mice. Values are mean fold change relative to WT ± SEM (Student’s t-test ; * p ≤ 0.05).

**
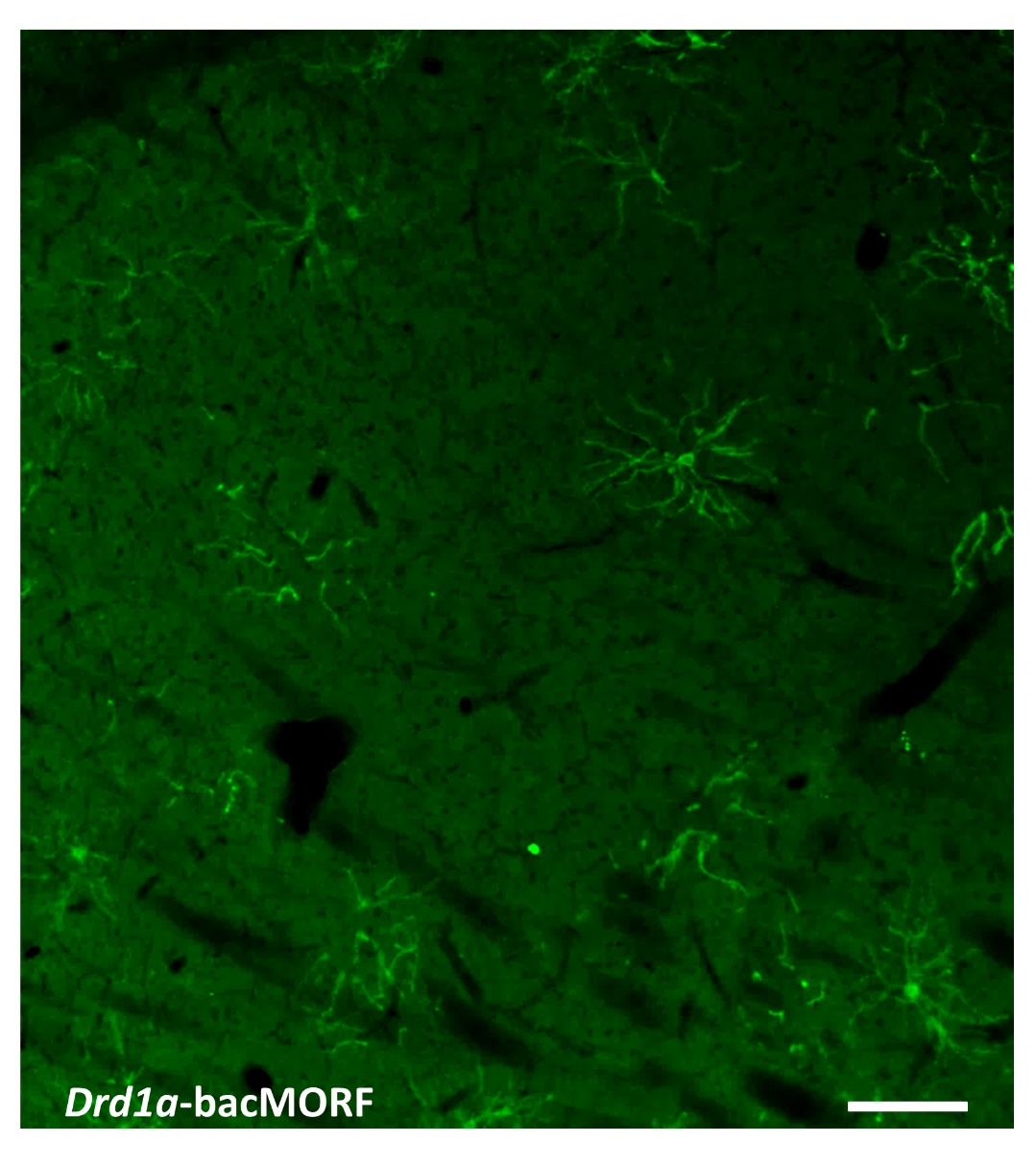
**

**Supplementary Figure S2. A second transgenic mouse line of D1-bacMORF-fGFP mice also confers sparse and stochastic labeling of D1-MSNs in striatum.** A representative image of sparse, fGFP labeled striatal D1-MSNs in this mouse line (scale bar= 50 μm).

**a b
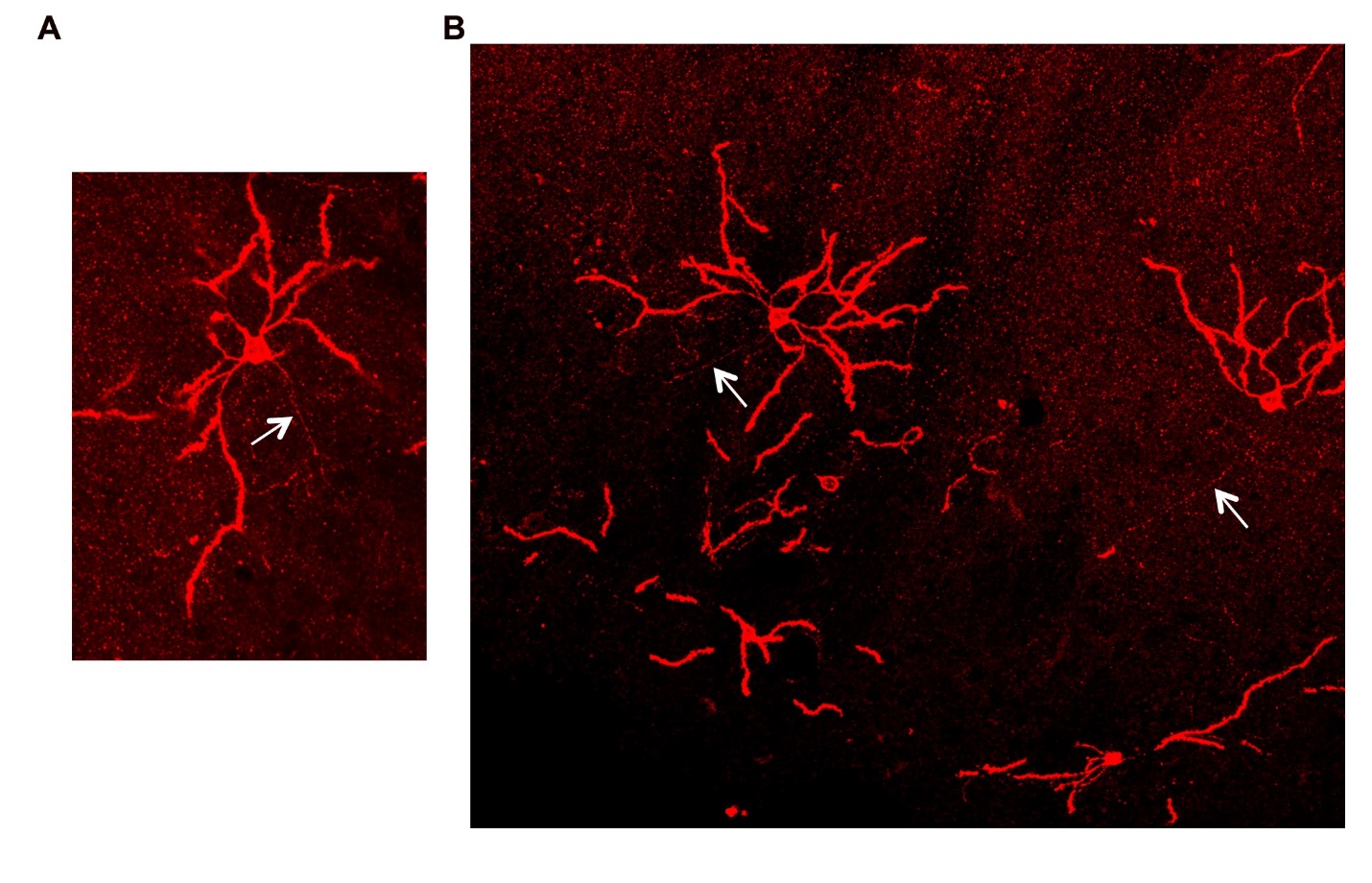
**

**Supplementary Figure S3. Drd1a-bacMORF mice can label proximal axonal segments in D1-MSNs.** A and B. representative confocal images of MORF-labeled D1-MSNs showing proximal axonal segments (white arrow) as well as dendrites with spines. Only a subset of labeled D1-MSNs show axonal labeling using our immunostaining protocol (see Experimental Procedure), and the labeling strength appears weaker than that for the dendrites.


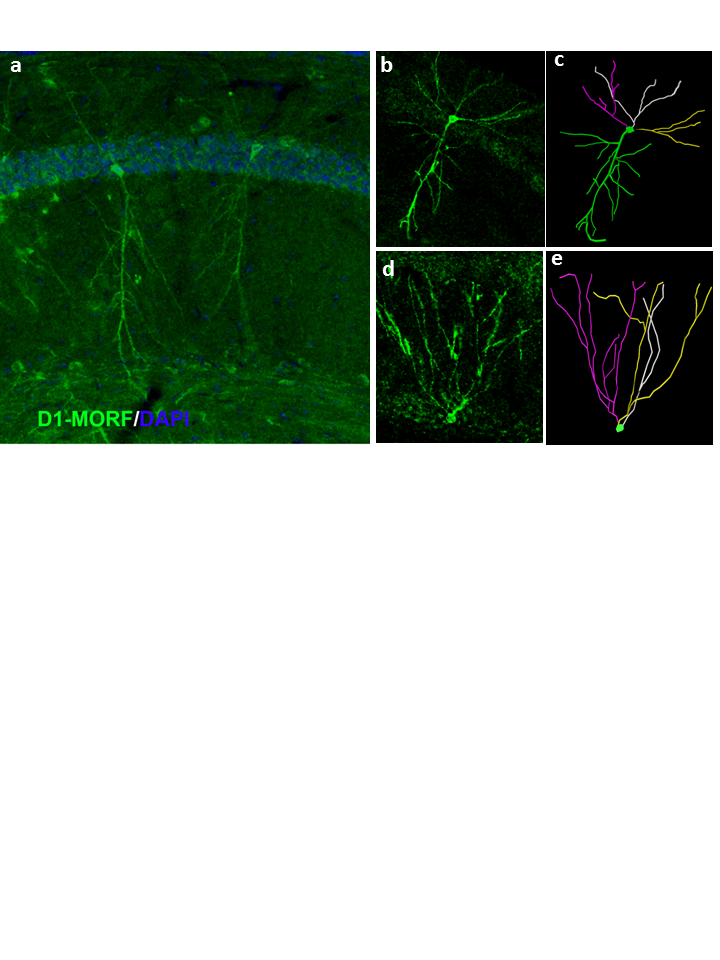


**Supplementary Figure S4. Drd1a-bacMORF Mice Show Sparse Labeling of Hippocampal Neurons.** (**a**) Sparse labeling of hippocampal pyramidal neurons in CA1 and CA2 regions. A projection of the stacked confocal images of a hippocampal pyramidal neuron (**b**) and its 3D reconstruction (**c**) in Drd1a-bacMORF mice, with apical dendrites labeled green. (**d, e**). Stacked confocal images of a single labeled hippocampal granule neuron in the dentate gyrus (**d**) and its 3D reconstruction (**e**).


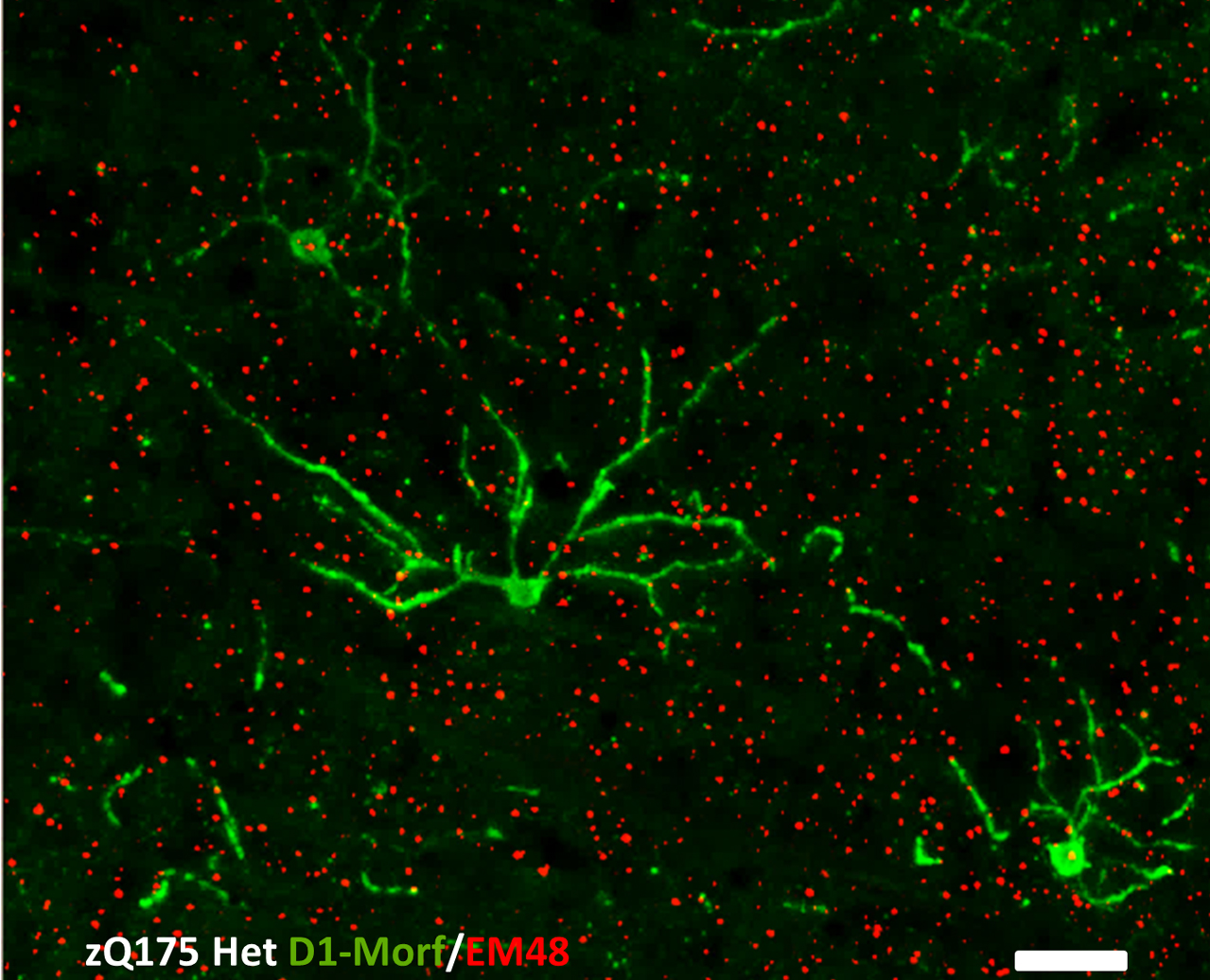


**Supplementary Figure S5. Using Drd1a-bacMORF mice to image both single D1-MSNs and mHtt aggregates.** *Drd1a*-bacMORF mice were crossed with an HD knock-in mouse model expressing full-length mutant Htt (mHtt) with 175Q from its endogenous locus. Double immunofluorescent staining was performed for aggregated mHtt (EM48) and MORF-labeled single D1-MSNs in zQ175 HD mice at 6-7m of age. MORF labeled D1-MSNs (in green fluorescence) were identified in the zQ175 heterozygous mice at 6-7 months of age (scale bar = 10 μm). EM48 positive mHtt aggregates are labeled in red fluorescence.
